# Detection and molecular analysis of *Campylobacter ureolyticus* in domestic animals

**DOI:** 10.1186/1757-4749-6-9

**Published:** 2014-04-16

**Authors:** Monika Koziel, Gerard D Corcoran, Roy D Sleator, Brigid Lucey

**Affiliations:** 1 Department of Biological Sciences, Cork Institute of Technology, Bishopstown, Cork, Ireland; 2 Department of Diagnostic Microbiology, Cork University Hospital, Wilton, Cork, Ireland

**Keywords:** *Campylobacter ureolyticus*, Emerging pathogen, Reservoirs, Domestic animals, Zoonosis

## Abstract

Previous studies showed the presence of *Campylobacter ureolyticus* in a large proportion of diarrhoeal samples from patients in Ireland. This emerging gastrointestinal pathogen was the second most common *Campylobacter* species detected in patients presenting with gastroenteritis, surpassed only by *C. jejuni*. However, the source of *C. ureolyticus* infections in humans remains unknown. The aim of this study was to investigate the presence of *C. ureolyticus* in a range of domestic animals. Over a period of 6 months, 164 samples collected from various domestic animals were tested using molecular method based on detection of the *C. ureolyticus* specific *hsp60* gene. These included canine faeces (n = 44), feline faeces (n = 31) and porcine faeces (n = 89). *C. ureolyticus* was detected in 32% (10/31) of feline faeces, 9% (4/44) of canine faeces and 18% (16/89) of porcine faeces. Random Amplified Polymorphic DNA (RAPD) analysis of *C. ureolyticus* isolates showed that an isolate from a cat is genetically similar to a strain isolated from a patient presenting with gastroenteritis.

This study reports the first detection and isolation of this organism in domestic animals in Ireland, with a potential source for human infection. Together with the previously reported detection of *C. ureolyticu*s in bovine samples, it is likely that this emerging pathogen has a zoonotic potential.

## Background

Infections with *Campylobacter* spp. account for a significant proportion of the reported cases of bacterial gastroenteritis worldwide [1].

Understanding the spread of campylobacter and its transmission from environmental sources to humans is essential for disease control and prevention. *Campylobacter* spp. have been detected in a variety of sources, such as wild birds and rivers [2,3]. However, campylobacteriosis is mostly a disease of zoonotic origin - inappropriate handling and consumption of undercooked, contaminated meat and other food products of animal origin, such as unpasteurised milk, are the most important risk factors for *Campylobacter* spp. infection [4]. While chickens and cattle are the most common animal reservoirs of *Campylobacter* spp, particularly *C. jejuni* and *C. coli*, other animals, such as pigs, sheep and dogs have also been linked to human infections [5-7].

Recently, molecular-based studies of the prevalence of various *Campylobacter* spp. in patients presenting with gastroenteritis in Southern Ireland, have identified *Campylobacter ureolyticus* as the second most common *Campylobacter* species, after *C. jejuni*[8]. Other studies have detected *C. ureolyticus* in patients suffering from diarrhoea [9,10], Crohn’s disease [11,12] and ulcerative colitis [13].

However, despite being detected in a large proportion of patients, the possible sources of human infections still remain unclear.

In this study we investigate the presence of *C. ureolyticus* in samples obtained from animals which were previously shown to be positive for *Campylobacter* species such as *C. jejuni* and *C. coli*, in order to elucidate possible routes of transmission of this emerging pathogen to humans.

## Results

From November 2012 to March 2013, 164 faecal samples were collected from domestic animals (dogs, cats and pigs). These were tested for *C. ureolyticus* with species-specific polymerase chain reaction (PCR) and the positive results were cultured. The results are presented in Table 1.

**Table 1:** Summary of PCR-positive results obtained throughout the study

**Sample source**	**PCR positive samples (%)**	**Culture positive samples (%)**
Porcine	16 (18%)	0/16 (0.0)
Canine	4 (9%)	0/44 (0.0)
Feline	10 (32%)	1/31 (3.2)

Of 89 porcine samples tested, 16 (18%) tested positive for the presence of *Campylobacter ureolyticus* (Table 1). In Farm 1, the majority of the *C. ureolyticus*-positive piglets were aged ≥2 months and a large proportion of piglets 6–9 days old was also positive for this organism. None of the 16 piglets aged 4–6 weeks tested positive for *C. ureolyticus*. Since no piglets younger than 2 months old were available in Farm 2 at the time of testing, no comparable data are available from this farm.

*C. ureolyticus* was also detected in pets in this study. A total of 32% of cat faecal samples and 9% of dog faecal samples were positive for this organism. These samples were collected from both local animals and animals in the care of two Cork veterinary hospitals.

**Figure 1:**
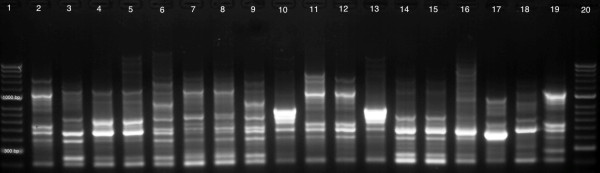
**1.5% agarose gel of RAPD profiles of *C. ureolyticus *isolates generated with (GTG)**_**5 **_**primer.** Lane 1: 100 bp molecular weight marker; Lane 2: CIT 001; Lane 3: CIT 002; Lane 4: CIT 003; Lane 5: CIT 004; Lane 6: CIT 005; Lane 7: CIT 006; Lane 8: CIT 007; Lane 9: CIT 008; Lane 10: CIT 009; Lane 11: CIT 010; Lane 12: CIT 011; Lane 13: CIT 012; Lane 14: CIT 013; Lane 15: CIT 014; Lane 16: DSMZ 20703^T^; Lane 17: CCUG 9510; Lane 18: CCUG 58468; Lane 19: CCUG 59897; Lane 20: 100 bp molecular weight marker.

All PCR-positive samples were inoculated on NAV medium. Among all 30 animal samples cultured, one strain of *C. ureolyticus* was isolated from cat faeces. To investigate the genetic similarity of this strain (CIT 012) to isolates obtained from patients’ faecal samples and culture collection strains, RAPD analysis with (GTG)_5_ primer was performed (Figure 1).

**Figure 2:**
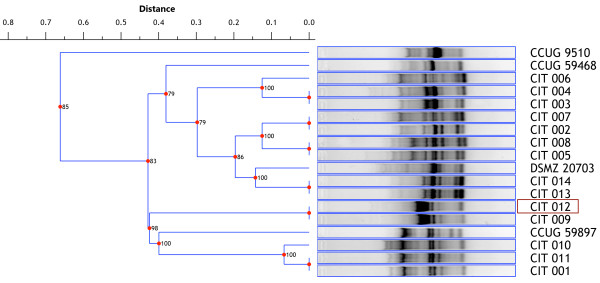
**Dendrogram of *C. ureolyticus *RAPD profiles.** Unweighted Pair Group Method with Arithmetic Mean (UPGMA) with a dice coefficient distance tree was generated with Phoretix 1D Pro software. Highlighted isolate (CIT 012) is the feline isolate.

Gel profiles were compared and clustered on the basis of pattern similarity, using Phoretix 1D Pro software, and the obtained dendrogram is presented in Figure 2. Feline isolate CIT 012 clustered closely with the CIT 009 isolate obtained from an elderly female suffering from gastroenteritis.

Antibiotic susceptibility testing (AST) with disc diffusion method showed that strain CIT 009 and CIT 012 are resistant to ciprofloxacin, nalidixic acid and trimethoprim.

## Discussion

The emerging role of *Campylobacter ureolyticus* as a potential cause of human gastrointestinal illness has been of interest to various research groups worldwide. However, reported isolations of *C. ureolyticus* from animals are very limited and mostly associated with endometrial infections in mares [14,15], potentially linked to infertility in these animals.

A previous study, performed by our research group, reported the presence of *C. ureolyticus* in a total of 13% of unpasteurised milk samples, collected across two different farms in Co. Cork, Ireland [16]. However, an investigation into a zoonotic potential of this emerging pathogen and its association with domestic animals, which are common transmission vectors for other *Campylobacter* species such as *C. jejuni*, has not yet been investigated. This study was undertaken to investigate the presence of *C. ureolyticus* in domestic and companion animals using molecular and traditional approaches.

Among 89 porcine faecal swabs collected, 18% (n = 16) tested positive for *C. ureolyticus* (Table 1). A similar proportion of *C.ureolyticus*-positive animals was observed across the two farm sites tested with 19% of pigs in Farm 1 and 17% of pigs in Farm 2 positive for this organism (Table 2).

**Table 2:** Summary of positive porcine faecal samples from the two farms sampled

**Number of positive samples (%)**				
**Farm site**	**Age of piglets**			
	**6–9 days**	**4–6 weeks**	**≥2 months**	**Total**
Farm 1	7/29 (24)	0/16 (0)	3/8 (38)	10/53 (19)
Farm 2	N/A	N/A	6/36 (17)	6/36 (17)
**Total**	4/29 (14)	0/16 (0)	9/44 (20)	**16/89 (18)**

The overall positive sample rate was similar for the two farms tested, despite the fact they were separated geographically. Interestingly, a similar study by Soultos *et al*. [17] investigating the prevalence of *Campylobacter coli* in piglets aged 3–66 days and their sows reported that a majority of piglets in their first days of life were colonised with *Campylobacter coli* genotypes resembling those isolated from the sow. These were often displaced by other genotypes in 3 month old piglets. Many of the piglets and sows carried multiple genotypes of *C. coli*[17]. Since no isolates were recovered from piglets in this study, no investigation into the genetic diversity of *C. ureolyticus* strains in piglets at different age was possible.

As no other study has investigated the prevalence of *C. ureolyticus* in porcine samples, no direct comparison of the levels found with these reported in this study is possible. However, other studies report significant rates of fastidious *Campylobacter* species, such as *C. concisus* (9%) or *C. helveticus* (6%) in porcine caecal content tested [18]. The rates reported in this study for *C. ureolyticus* are comparable with those reported for other fastidious species [18]. The high prevalence of *C. ureolyticus* in the porcine gastrointestinal tract, highlights the potential for zoonotic transmission, especially if abattoir hygiene was not maintained at acceptable levels. This could be of particular importance for retail products, such as cured or smoked ham and bacon, which do not undergo extensive heat treatment, which would otherwise significantly decrease the number of viable bacteria in foodstuffs. However further studies are needed to determine whether consumption of such foods increases the risk for *C. ureolyticus* infection in humans.

*Campylobacter ureolyticus* was also detected in faecal samples collected from cats and dogs. These included samples collected from companion household animals as well as animals in veterinary care. A total of 9% of canine and 32% of feline samples tested positive for the presence of *C. ureolyticus*. Metagenomic studies carried out on cats, report percentages of *Campylobacter* spp. as high as 10% [19]. Moreover, *Campylobacter* spp. of potential clinical relevance, other than *C. jejuni* and *C. coli*, such as *C. upsaliensis*, have been shown to be present in dog faeces [6]. PCR detection rates of *Campylobacter* spp. from dog faeces are highly variable (58–97%) depending on the study, geographical location and methodology used [20].

Various factors appear to influence the level of *Campylobacter* spp. faecal shedding in cats and dogs. These include the age of the animal, with puppies < 6 months and cats <36 months more frequently positive for *Campylobacter* spp. [21-23]; housing conditions, with dogs housed in kennels or shelters and outdoor cats with no access to litter trays, more at risk of campylobacter infections [23,24]; and dogs’ diet, with a higher risk of campylobacter infection for animals fed a homemade food diet [25].

It has been shown that strains isolated from patients with *Campylobacter jejuni* infections, particularly young children, and their pets are genetically similar, confirming the possibility of zoonotic transmission of this genus [26-28].

This finding is of potential importance for understanding the possible sources of *C. ureolyticus* infections in humans. The majority of patients suffering from gastroenteritis in which *C. ureolyticus* was detected, were reported to be children <5 years old or adults >70 years old [29]. In this study, we have shown that the *C. ureolyticus* strain isolated from a cat’s faeces is genetically similar to the isolate CIT 009 cultured from the diarrhoeal stool of an elderly patient (Figure 2). RAPD profiles of these two isolates were identical and clustered together with other strains isolated from the faeces of patients aged 83 (CIT 001), 81 (CIT 010) and 3 years old (CIT 011) as well as an isolate recovered from the blood of an elderly patient (78 years).

The isolation of *Campylobacter ureolyticus* from animal faeces is difficult, with an isolation rate of 3.2% from PCR-positive cat samples and zero from pig and dog samples. The difficulty in isolation can be partially attributed to a lack of selective medium for *C. ureolyticus*. This organism is sensitive to many antibiotics commonly used in media supplements for *C. jejuni* or *C. coli*, such as cefoperazone or cycloheximide. Moreover, the fastidious nature of the species together with an optimal growth temperature of 37°C, rather than 42°C, make it more difficult to isolate than, for example, *C. jejuni*, especially from samples, such as faeces, which have a large number of competing microorganisms.

Culturing of *Campylobacter spp*. is problematic even for species such as *C. jejuni* or *C. coli*, for which use of selective temperature, selective media and well established protocols is possible. It has been previously shown that among diarrhoeal samples from patients, as much as 50% were not cultured, despite being determined to be *Campylobacter jejuni* and *C. coli*[30,31]. Therefore, the prevalence of fastidious *Campylobacter* species is even more likely to be underestimated when traditional culture methods, targeting mostly thermophilic species, are employed.

The difficulty in culturing campylobacters in general, may also be a attributed to the presence of organisms in a viable but nonculturable state. It has been shown that campylobacter cells which enter this state, cannot be cultured on artificial media, despite remaining virulent and capable of invading intestinal cells [32].

Molecular based detection offers an alternative, allowing us to circumvent the problems facing traditional culture based approaches. Therefore, despite a failure to grow pathogenic *Campylobacter* spp., their detection with molecular methods is still likely to be of clinical significance.

## Conclusion

The effect of *C. ureolyticus* infections on animal health has, to date, not been investigated in any great detail. All stools samples collected throughout this study, were non-diarrhoeal, therefore no comparison of *C. ureolyticus* rates in healthy and sick animals was possible.

Nevertheless, this is the first study reporting the presence of *C. ureolyticus* in samples collected from domestic animals. Moreover, comparison of genetic profiles of isolates from animal and human origins shows the potential involvement of pets in *C. ureolyticus* infection, highlighting its potential for zoonotic transmission.

## Methods

### Sample collection

Faecal samples from asymptomatic pigs (n = 89), dogs (n = 44) and cats (n = 31) were collected between November 2012 and March 2013.

Porcine samples were obtained from piglets age 6–9 days old (n = 29), 4 weeks old (n = 16) and 2 months old (n = 44) from two separate pig farms in Southern Ireland, located approximately 20 km apart. Piglets were swabbed using sterile swabs (Deltalab) and the faecal samples were placed in 15 ml conical tubes (Corning) filled to the top with Bolton broth (Oxoid) with NAV supplement [33] which consisted of (l^-1^): 2 g sodium formate (Fluka), 3 g sodium fumarate (Fluka), 10 mg of amphotericin B, 10 mg nalidixic acid and 20 mg of vancomycin (all supplied by Sigma Aldrich).

Canine and feline samples were collected from local domestic animals and animals cared for in two separate veterinary hospitals in Co. Cork. Fresh faecal matter was placed in 25 ml centrifuge tube (Sarstedt) filled with Bolton Broth with NAV supplement.

### Sample preparation

All collected samples were immediately closed tightly (to create anaerobic conditions) and transported to the laboratory for incubation.

Samples were incubated aerobically at 37°C for a minimum of 7 days. Following incubation, samples were tested for the presence of *C. ureolyticus* using PCR. Remaining enriched broths were kept at 37°C for culture of any positive results following molecular analysis.

### Molecular detection

Following incubation, 2 ml of enriched sample was taken for molecular analysis. Samples were centrifuged at 17,000 × g for 1 minute and bacterial DNA from the pellet was extracted with QIAamp DNA Stool Mini Kit (Qiagen). The presence of *C. ureolyticus* was investigated as previously described [16]. In brief, *C. ureolyticus*-specific primers targeting the *hsp60* gene of this organism [34] were used for analysis of the samples. Potential PCR inhibition by the sample was monitored by including an internal amplification control (IAC) in every PCR reaction.

All PCR reactions consisted of 1U of HotStarTaq Plus DNA Polymerase (Qiagen), 1 μM of each primer (Eurofins, MWG Operon), 0.2 μM of each dNTPs (Sigma Aldrich) and 100 ng of DNA template. The *C. ureolyticus* DSMZ 20703 type strain was used as a positive control for both extraction and PCR amplification. PCR conditions were as suggested by the *Taq* manufacturer and the annealing temperature was set at 58°C for 1 minute. Amplicons were visualised on 1.5% agarose gels and the results were interpreted on the basis of presence/absence of a band of 429 bp for *C. ureolyticus* positive samples and IAC amplification (196 bp) for *C. ureolyticus* negative samples. Positive amplicons were purified using QIAquick PCR Purification Kit (Qiagen) and PCR positive products were sequenced (GATC Biotech) using the *hsp60* primers to ensure the validity of the positive results.

### Culture methods

Samples were cultured immediately, once presumptive positive results from the PCR were obtained, to attempt recovery of *C. ureolyticus*. Enriched samples were inoculated onto NAV agar [33], consisting of (l^-1^) 46 g of Anaerobe Basal Agar (Oxoid), 10 g Agar (Sigma Aldrich) and NAV supplement described previously. Each sample was inoculated in triplicate and incubated anaerobically using AnaeroGen 2.5 L gas packs (Oxoid) at 37°C for up to 14 days. Plates were checked during that period at regular intervals (3–4 days) for presumptive *C. ureolyticus* colonies: flat, translucent, spreading colonies. Colony PCR with *C. ureolyticus* - *hsp60* primers was performed on putative colonies. All PCR positive isolates were sent for 16S *rRNA* sequencing using fD1 and rP1 primers [35] to confirm their identity.

### RAPD analysis and comparison with human isolates

*Campylobacter ureolyticus* strains isolated from animals were compared with human isolates obtained from patients presenting with gastroenteritis, healthy controls and strains available from culture collections. Details of the isolates are shown in Table 3.

**Table 3:** Summary of the strains used in this study

**Strain**	**Gender (Age in years)**	**Sample source**	**Medical history**
CIT 001	Male (83)	Human faeces	Long term hospital stay
CIT 002	Female (84)	Human faeces	End stage chronic renal disease
CIT 003	Male (65)	Human faeces	Nursing home resident. Recurring diarrhoea
CIT 004	Female (3)	Human faeces	Admitted to the hospital with diarrhoea and vomiting. Cryptosporidium oocyst detected.
CIT 005	Female (3)	Human faeces	Positive for cryptosporidium oocysts
CIT 006	Female (71)	Human faeces	Long term hospital stay
CIT 007	Female (84)	Human faeces	End stage chronic renal disease. Recurring diarrhoea
CIT 008	Female (55)	Human faeces	Hepatic cirrhosis/diabetes
CIT 009	Female (83)	Human faeces	Nursing home resident
CIT 010	Female (81)	Human faeces	Nursing home resident
CIT 011	Female (3)	Human faeces	N/A
CIT 012	Female (2)	Cat faeces	asymptomatic, healthy (This study)
CIT 013	Female (26)	Human faeces	asymptomatic, healthy
CIT 014	Male (33)	Human faeces	asymptomatic, healthy
DSMZ 20703^T^*	Female	Amniotic fluid	N/A
CCUG 9510*	Male (22)	Penis wound	N/A
CCUG 59468*	Female	Vagina	N/A
CCUG 59897*	Unknown (78)	Human blood	N/A

*Strains were obtained from Culture Collection, University of Göteborg, Sweden (CCUG) and Leibniz Institute DSMZ-German Collection of Microorganisms and Cell Cultures, Braunschweig, Germany.

To obtain RAPD profiles, PCR reactions were carried out with (GTG)_5_ primer [36] at a final concentration of 1 μM, 1U GoTaq DNA Polymerase (Promega), 0.2 μM of each dNTPs and 100 ng of template DNA. Thermal cycling conditions were as follows: 1 initial denaturation cycle at 94°C for 5 min, followed by 34 cycles of denaturation at 94°C for 2 min, annealing as described later and extension at 72°C for 2 min followed by the final extension step at 72°C for 5 min. To improve reproducibility, the first 4 cycles were performed at lower stringency, at annealing temperature of 36°C for 2 min, followed by 30 cycles at 50°C for 1 min. PCR amplicons were separated on 1.5% agarose electrophoresed at 70 V for 90 min. The gels were stained with ethidium bromide and visualised with UV transluminator (UVP, Cambridge, UK). Gels were analysed using Phoretix 1D Pro software (TotalLab) and similarities of RAPD fingerprints of all isolates were illustrated as clusters based on the banding patterns.

Antibiotic susceptibility of isolated CIT 009 and CIT 012 was determined using the disc diffusion method with the following discs tested: ciprofloxacin (30 μg), nalidixic acid (30 μg) and trimethoprim (5 μg). Both strains were susceptible to tetracycline (20 μg), erythromycin (15 μg), gentamicin (10 μg) and cefoperazone (30 μg) (all discs were supplied by Oxoid). Bacterial suspension were adjusted to 0.5 McFarland in PBS and spread onto Mueller Hinton agar (Sigma) supplemented with 5% defibrinated horse blood (TCS Biosciences). Plates were incubated anaerobically for 48 hours; in case of insufficient growth, plates were re-incubated for further 24 hours. Zone diameters were interpreted accordingly to recommendations of the European Committee on Antimicrobial Susceptibility Testing [37], the British Society for Antimicrobial Chemotherapy [38] and breakpoints used for *Campylobacter* spp. in other studies [39]. In the absence of such information, breakpoints were decided by authors as susceptible ≥10 mm and resistant ≤10 mm.

## Abbreviations

PCR: Polymerase Chain Reaction; RAPD: Random Amplified Polymorphic DNA; MLST: Multilocus Sequence Typing; NAV: Nalidixic acid, amphotericin, vancomycin; UPGMA: Unweighted Pair Group Method with Arithmetic Mean; N/A: Not applicable.

## Competing interests

The authors declare that they have no competing interests.

## Authors’ contributions

MK designed the study, carried out the experimental work, performed the analysis, and drafted the manuscript. RDS and BL designed and coordinated the study, and edited the manuscript. GDC edited the manuscript. All authors read and approved the final manuscript.
